# Perspectives on the Influence of Crystal Size and Morphology on the Properties of Porous Framework Materials

**DOI:** 10.3389/fchem.2021.772059

**Published:** 2021-11-11

**Authors:** Claire L. Hobday, Simon Krause, Sven M. J. Rogge, Jack D. Evans, Hana Bunzen

**Affiliations:** ^1^ Centre for Science at Extreme Conditions and EaStCHEM School of Chemistry, The University of Edinburgh, Edinburgh, United Kingdom; ^2^ Nanochemistry Department, Max Planck Institute for Solid State Research, Stuttgart, Germany; ^3^ Center for Molecular Modeling (CMM), Ghent University, Ghent, Belgium; ^4^ Centre for Advanced Nanomaterials and Department of Chemistry, University of Adelaide, Adelaide, SA, Australia; ^5^ Chair of Solid State and Materials Chemistry, Institute of Physics, University of Augsburg, Augsburg, Germany

**Keywords:** crystal size, metal-organic frameworks, soft porous crystals, mechanical stability, adsorption, molecular simulation, drug delivery

## Abstract

Miniaturization is a key aspect of materials science. Owing to the increase in quality experimental and computational tools available to researchers, it has become clear that the crystal size and morphology of porous framework materials, including metal-organic frameworks and covalent organic frameworks, play a vital role in defining the physicochemical behaviour of these materials. However, given the multiscale and multidisciplinary challenges associated with establishing how crystal size and morphology affect the structure and behaviour of a material–from local to global structural modifications and from static to dynamic effects–a comprehensive mechanistic understanding of size and morphology effects is missing. Herein, we provide our perspective on the current state-of-the-art of this topic, drawn from various complementary disciplines. From a fundamental point of view, we discuss how controlling the crystal size and morphology can alter the mechanical and adsorption properties of porous framework materials and how this can impact phase stability. Special attention is also given to the quest to develop new computational tools capable of modelling these multiscale effects. From a more applied point of view, given the recent progress in this research field, we highlight the importance of crystal size and morphology control in drug delivery. Moreover, we provide an outlook on how to advance each discussed field by size and morphology control, which would open new design opportunities for functional porous framework materials.

## Introduction

Reports of novel crystalline materials, including metal-organic frameworks (MOFs) and covalent organic frameworks (COFs), are ubiquitous in the literature. Several studies have highlighted that crucial material properties of these systems can strongly differ in response to seemingly small changes to the chemical composition. For example, lattice defects can produce unexpected catalytic activity, adsorption properties, and mechanical stability (Cheetham et al., 2016). These defects impact the framework composition and can be used to engineer framework materials that deviate from their ideal framework behaviour. Incredibly, there is an increasing number of studies that report the manipulation of framework properties by simply modifying the size and morphology of the crystals. This opens a wealth of new possibilities to material engineering and design, and additionally, opens questions to how the crystal size and morphology affect important properties?

In this perspective, we outline the current progress to understanding and harnessing crystal size and morphology in relation to mechanical properties, adsorption-induced stress, phase stability, computational approaches, and biomedical applications.

## Mechanical Properties

The field of material science has long understood the importance that particle size can impose on the mechanical properties of materials, notably, in the areas of activated carbons (Saeidi and Lotfollahi, 2015), magnetorheology (De Vicente et al., 2011) and composite materials (Lim et al., 2010). As the field of MOFs and COFs begins to explore a range of particle sizes, from the creation of monoliths (Tian et al., 2015; Widmer et al., 2018) to nanoparticles (Osterrieth and Fairen-Jimenez, 2021), we are beginning to see the effect this can have on mechanical properties of the materials.

The preparation and understanding of monolithic structures are important for the industrial development of MOFs, as the as-synthesised form of a microcrystalline powder causes difficulty in chemical processing such as low volumetric efficiency, poor heat and mass transfer and high pressure drop when compressed in a flat bed. Monoliths provide a way of shaping the MOF into a macroscopic structure which has improved or more homogeneous mechanical properties and, most importantly, improved application in industrial settings. MOF monoliths were first prepared as a way of expanding the volumetric storage capacity in MOF-177 (Zacharia et al., 2010) and MIL-101 (Oh et al., 2014). In both cases this was achieved, but due to the compression involved in densifying the samples, their gravimetric capacity was greatly reduced. This was because large pressures (up to 1 GPa) were used on the samples, causing amorphisation and collapse of the pore structure. Other methods employed to build monoliths involved the addition of binders, additives and extrusion all of which impacted on the internal pore volume (Küsgens et al., 2010). However, if monoliths are prepared under milder conditions, like that of Fairen-Jimenez and co-workers (Tian et al., 2015; Connolly et al., 2019), the internal porous structure can be maintained. They exemplified this process by first creating monoliths of ZIF-8, which had higher Young’s modulus and hardness than single crystals of ZIF-8. They attributed these improved mechanical properties to the mild drying conditions, which facilitated the continuation of polymerisation between charged ZIF-8 particles and unreacted starting material. They then refined the process to apply to many archetypal MOFs, such as UiO-66 (Connolly et al., 2019) and HKUST-1 (Tian et al., 2018). This is an important step in the self-shaping of MOFs, and provides a blueprint to create tunable mesoporosity, improved mechanical properties and densification of the materials whilst maintaining the internal microporous structure.

While the development of monoliths will improve the use of framework materials in large process chemistry, nanoparticles offer novel properties as outlined in *The Importance of Crystal Size and Morphology in Drug Delivery*. Downsizing to the nanoscale increases the surface to volume ratio and as a result, nanocrystals of MOFs have exhibited different mechanical and structural properties to their microcrystalline counterparts. Curious examples include ZIF-8 which as a monolith exhibited a larger Young’s modulus (7.7 GPa) than standard microcrystals (3.4 GPa) (Tian et al., 2015); this trend continues into the nanoscale where the Young’s modulus is further reduced (2.3 GPa) (Tiba et al., 2019). This increase in flexibility for ZIF-8 at reduced particle size has important ramifications for its other physical properties such as its swing-effect, whereby the methylimidazolate linkers rotate upon the uptake of guests, in order to maximise the pore volume. The swing effect was first recorded in a high-pressure crystallographic experiment where methanol was forced into the pores of a large crystal of ZIF-8 (300–400 µm) at the GPa regime (Moggach et al., 2009). The same swing effect was recorded on a microcrystalline powder of ZIF-8 at orders of magnitude lower (2 kPa) (Fairen-Jimenez et al., 2011), however, upon downsizing the crystal further the same trend is not observed. A comprehensive study by Denayer and co-workers demonstrated this effect by recording the decreasing swing effect pressure upon increasing the size of ZIF-8 particles between 0.060 and 88 µm (Tanaka et al., 2015). This difference has been attributed to the decreased number of methylimidazolate, as well as a decrease in mass transfer due to the larger surface barrier in smaller crystals.

The downsizing effect of MOFs has long been discussed if it can be applicable to shape-memory materials (Sakata et al., 2013). This relies on the creation of a metastable state, such like that of swing-open phase of ZIF-8, to be retained upon the removal of the guest, while this is not the case for ZIF-8, there is still a large hysteresis for smaller ZIF-8 particles. Whilst we move towards a greater understanding of MOFs at the nanoscale, and the different properties which persist at this scale, there is still much work to be done. Later chapters explore how in tandem with modified mechanical properties at the nanoscale, other properties emerge such as unique adsorption or shape memory effects. This is an exciting time for the field, where we recognise particle size as an additional variable in which to tune MOF properties, which opens up new avenues for MOFs such as the development of nanoscale sensing and chemoswitching.

## Adsorption-Induced Stress

Adsorption-induced deformations of crystalline porous framework materials are fascinating processes with strong implications for many important such as storage, separation or sensing of gases and gas mixtures (Gor et al., 2017). The deformation of a porous host by interactions with a fluid guest has been known for centuries but recently gained attraction (Gor et al., 2017). The recent run for enhanced porosity and surface area has yielded porous solids with reduced wall thickness and enhanced mechanical instabilities. In addition, the synthesis of crystalline nanoporous frameworks resulted in ordered structures with detailed molecular design allowing to study and tune mechanical properties of the porous host, as discussed in the previous section. But adsorption-induced transformations of the porous host is a two-sided process: the mechanical stability of the framework is balanced against the magnitude of adsorption-induced stress which can be considered an internal force directed either towards expansion or contraction of the lattice. To observe adsorption-induced structural deformations the stress level has to overcome the activation barrier of structural deformation (Neimark et al., 2010). This will result in hysteresis in the adsorption isotherm and other adsorption phenomena such as gating, breathing or negative gas adsorption. As discussed above, crystal size is a critical parameter that influences the mechanical properties (Tian et al., 2015) of nanoporous framework materials beyond their structure and molecular composition. But what about the impact on the adsorption properties and stress levels?

There are two prototypical flexible framework systems with similar cubic symmetry that have been studied extensively with respect to crystal size phenomena, namely ZIF-8 and DUT-49. ZIF-8 is a microporous MOF that shows gate-opening transitions with minor expansion in unit cell dimension (Fairen-Jimenez et al., 2011). DUT-49 is a mesoporous MOF which exhibits breathing transitions and large successive contraction and expansion of the unit cell volume as a response to adsorption (Krause et al., 2016). Crystal-size dependent studies from Tanaka et al. (Tanaka et al., 2015) as well as Krause et al. (Krause et al., 2018) show that this factor can strongly influence the adsorption behaviour and adsorption-induced flexibility of the materials using the example of nitrogen adsorption at 77 K. In ZIF-8 the gas pressure of nitrogen required to induce gate opening is found to be shifted towards higher pressures for smaller crystals. In DUT-49 adsorption-induced structural contraction is found to be absent for samples with mean crystal sizes below ca. 1 µm. In both cases this behaviour is primarily associated with the stiffening of the material and enhanced activation barriers for structural transitions in the solid state (discussed in *Mechanical Properties* and *Altering the Phase Stability in Soft Porous Crystals Through Crystal Size Engineering*). However, the change in adsorption properties with respect to adsorption-induced stress as a function of crystal size has not been discussed in detail.

Nitrogen adsorption isotherms of downsized crystals of ZIF-8 at 77 K show interesting behaviour (Tanaka et al., 2015). At low pressures the isotherms overlap, at intermediate pressures a decrease in uptake for smaller crystals is found, and at larger pressures, close to saturation, a flat plateau for large crystals and a strong increase in uptake for smaller crystals is observed. Zhang et al. were able to reproduce this behaviour experimentally and theoretically by grand canonical Monte Carlo (GCMC) simulations on a perfect crystal lattice and a nanoscopic crystal of ZIF-8 (Zhang et al., 2014). In these simulations they were able to determine the contribution of adsorption in the crystal surface on the one hand and the crystal interior on the other hand to the overall adsorption isotherm. They showed that the adsorption interactions in the microporous interior are enhanced by ∼1.5 kT relative to the interfacial adsorption process.

The ratio of external surface area to internal crystal volume increases with decreasing crystal size. However, in porous solids external surface area is more than just the terminating atoms or molecules, which are found to only play a minor role (Zhang et al., 2014). The pore structure and pore diameters on the crystal exterior are impacted to a much stronger extent, penetrating into the interior of the crystals, resulting in an interfacial region of several unit cells of nm thickness. The relative volume of this interfacial region will drastically increase with decreasing crystal size, compared to the volume of the crystal interior. Adsorption in this interfacial region is generally negligible featuring a lower enthalpy of adsorption compared to that of the interior, however, at a certain size in the nanometer range this region becomes the dominating phase of the material. In the case of ZIF-8 this shifts the gate-opening transition pressure towards higher pressures. In addition, surface blockage and pore network blockage of the interior are identified as contributing factors (Sharma et al., 2020).

A similar behaviour was observed in DUT-49: adsorption isotherms of smaller crystals showed lower uptake at intermediate pressures and were found to no longer exhibit adsorption-induced contraction. As such, the lack of adsorption interactions and for that matter adsorption-stress reduces the thermodynamic driving force for adsorption-induced structural transitions in nanocrystals. This reduction in adsorption interactions upon crystal downsizing was experimentally demonstrated by ^129^Xe nuclear magnetic resonance (NMR) experiments in which the chemical shift of Xe was found to decrease during adsorption in DUT-49 with smaller crystal sizes (Krause et al., 2020). This study also highlights the effect of lattice defects in the crystal interior and finds that only a very large abundance of structural defects will have a significant impact on the adsorption stress, rendering the crystal interior similar to the surface interface.

Although these few studies provide an indication for a mechanism that governs the effect of crystal size on adsorption-induced deformations and although they established theoretical as well as experimental tools to study it, many open questions remain. How can adsorption stress be quantified experimentally and computationally? How is adsorption stress correlated with pore geometry and size, temperature, pressure, and physical nature of the gas? How and to what extent are these parameters impacted by the crystal size? A recent review by Ehrling et al. discusses the crystal size effects in switchable porous frameworks and provides an overview of known studies on materials other than DUT-49 and ZIF-8 (Ehrling et al., 2021). Nevertheless, in current studies the focus is too often put on discussing the effects with respect to the material rather than investigating the whole system which involves changes in the fluid phase and adsorption process as well. A recent study demonstrates that ZIF-8 becomes more flexible with decreasing crystal size by probing micro and nanocrystals with nanoindentation (Tiba et al., 2019). The fact that this trend is opposite to the evolution of adsorption-induced stress shows how important a global analysis of these effects with respect to mechanical properties of the host and adsorption properties of the fluid as a function of crystal size are.

We envision that an extension of combined experimental and theoretical approaches, first on model compounds such as ZIF-8 and DUT-49, later on a wider scope of dynamic porous solids, will provide more fundamental mechanisms that will ultimately allow to design such effects. We furthermore consider it crucial that aspects of crystal size are being documented in future studies to allow for a wider set of accessible data.

## Altering the Phase Stability in Soft Porous Crystals Through Crystal Size Engineering

Many frameworks can undergo large structural modifications, far beyond the elastic response discussed in *Mechanical Properties*, in response to external stimuli, such as gas sorption, photochemical triggers, and changes in temperature or pressure (Horike et al., 2009; Schneemann et al., 2014; Coudert, 2015). In the most extreme cases, the so-called soft porous crystal (SPC) even morphs between different phases under well-defined thermodynamic conditions. This dynamic nature opens the possibility to a whole range of dedicated applications, varying from sensing (Zhang et al., 2018) over energy storage and conversion (Yot et al., 2014; Sun et al., 2021) to biomedicine (Zhou et al., 2021) (see also *The Importance of Crystal Size and Morphology in Drug Delivery*). As a consequence, extensive efforts have been undertaken to control this stimuli-responsiveness through chemical modifications that alter the long-range interactions at play in these materials, allowing one to precisely engineer under which external triggers the material’s flexibility comes to expression.

In the last decade, it has become clear that, next to these chemical modifications, also the crystal size is a highly tuneable control parameter that substantially affects the flexibility and even phase stability of SPCs. Sakata et al. demonstrated that the guest-induced transition between an open pore and a closed pore phase in microsized pillared-layered shape-memory materials can be completely suppressed, in favor of the retention of the open pore phase upon desorption, once the crystal is downsized to critical dimensions in the range of a few tens of nanometers (Sakata et al., 2013). They found this to be a gradual effect: for intermediate crystal sizes, the guest-induced flexibility is only partially suppressed. This crystal size effect also alters the gas sorption characteristics of the material, with smaller mesosized crystals showing an increase in gate-opening pressure and adsorption/desorption hysteresis compared to standard microsized crystals.

**FIGURE 1:**
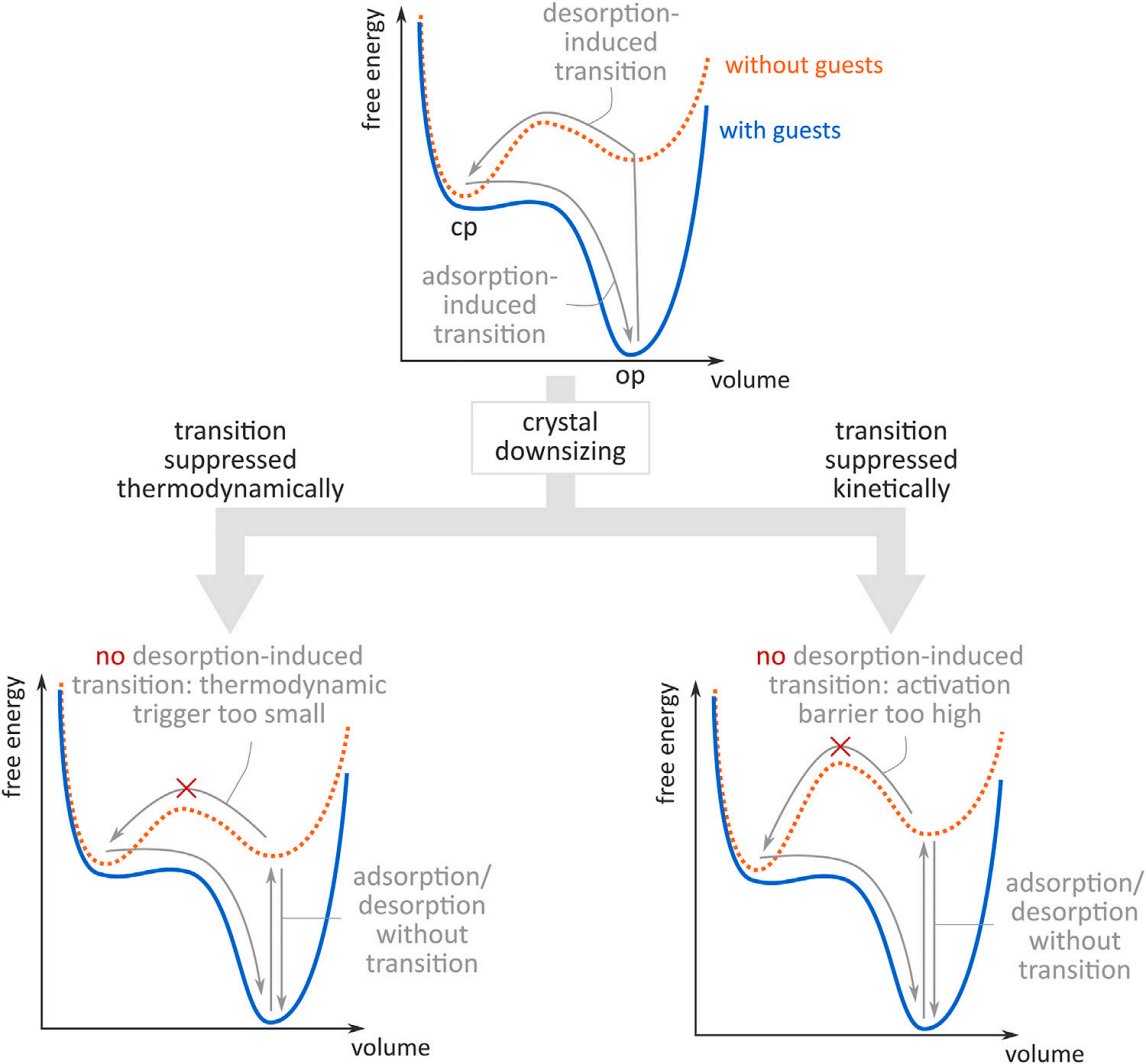
Schematic illustration of the free energy profiles with (orange) and without (blue) guests in a SPC. Top: guest adsorption/desorption induces a phase transition between an open pore (op) and a closed pore (cp) state. Bottom: upon crystal downsizing, the op-to-cp transition is suppressed, either thermodynamically due to too small a free energy difference between the op and the cp state **(left),** or kinetically due to too high an activation barrier between the op and cp state **(right).** Figure inspired by (Sakata et al., 2013).

Shape-memory effects have been realised for some MOFs upon downsizing, e.g., Kitagawa demonstrated this in 2013 with Cu_2_(bdc)_2_(bpy) (Sakata et al., 2013), other examples include X-pcu-3-Zn-3i (Shivanna et al., 2018) and DUT-8 (Miura et al., 2017). Importantly, Sakata et al. explored two possible explanations for this altered flexibility in mesosized crystals (Sakata et al., 2013), which are schematically illustrated in Figure 1. From a thermodynamic point of view, the increased surface-to-volume ratio in mesosized crystals may render the guest-free OP phase energetically more competitive compared to the guest-free CP phase thanks to the surface enthalpy contribution, reducing the thermodynamic driving force for the transition (bottom left panel of Figure 1). However, the suppressed phase transition could also be explained from a kinetic point of view. In this viewpoint, defect sites are thought of as preferential nucleation sites for phase transitions, as they tend to lower the lower the free energy barrier for transition, as was later computationally observed for other flexible materials (Rogge et al., 2019). As smaller mesosized crystals are assumed to contain fewer defect sites, crystal downsizing could increase the activation barrier between both phases (bottom right panel of Figure 1). This kinetic effect would also explain why the increase in sorption hysteresis was not accompanied by a modification of the center of the hysteretic loop upon downsizing in the aforementioned material.

Since then, the effect of crystal size on the phase stability and flexibility of SPCs has been observed in several other materials. Downsizing DUT-8 crystals below 500 nm was found to completely suppress the flexibility in these otherwise flexible crystals, while the flexibility was only partially suppressed for DUT-8 crystal sizes between 500 nm and 1 μm (Miura et al., 2017; Ehrling et al., 2019). Similarly, submicrometer ZIF-8 crystals are substantially more rigid than their microsized counterparts, as exemplified by the larger gate-opening pressure and the increased hysteretic loop upon nitrogen sorption for submicrometer crystals (Zhang et al., 2014; Tian et al., 2016). For these ZIF-8 samples, also the center of the hysteretic loop was encountered at higher pressures upon downsizing, in contrast to the work of Sakata et al. (Sakata et al., 2013), which may indicate that downsizing ZIF-8 also alters the thermodynamic stability of the different phases. By constructing a core-shell model, Zhang et al. could explain this effect based on the increased surface-to-volume ratio in smaller crystals, as this surface region shows a reduced uptake and hence a smaller driving force for guest-induced gate-opening (Zhang et al., 2014). In the same vein, the aforementioned negative gas adsorption in DUT-49 could be systematically suppressed upon downsizing, up to a level that no negative gas adsorption occurred for crystals smaller than about 1 μm (Krause et al., 2018; Krause et al., 2020). Next to nanocrystals, this suppression of flexibility upon crystal downsizing could also be observed in surface-anchored SPCs when the SPC film thickness is sufficiently reduced (Wannapaiboon et al., 2019).

In contrast to the aforementioned examples in which the SPC becomes more rigid upon downsizing, downsizing SPC nanocrystals could also promote flexibility in some limited cases, as shown for instance in amino-functionalized MIL-53(Al) samples (Kundu et al., 2019) and ZIF-7 (Sun et al., 2018), which was hypothesized to occur because of the increased flexibility of their surface region. Moreover, a remarkable example is that of Fe(py)_2_[Pt(CN)_4_], a 2D interdigitated non-porous framework, which when reduced down to a thin film of 16 nm, can transform to an OP phase through interaction with just the vapour of guest molecules which cause the interdigitated structure to be prised apart (Sakaida et al., 2016). Finally, it should be noted that the crystal size of a material is not a static property, but can hugely vary with the particle lifetime. For instance, Bon et al. demonstrated that the gate-opening pressure in DUT-8(Ni) and SNU-9 substantially increased upon repeated adsorption/desorption cycles, a direct result of the decrease in particle size upon cycling caused by the adsorption stress (Bon et al., 2015).

While crystal size engineering may hold great promise to tune the flexibility in SPCs, major advances in this field require a thorough understanding of how crystal size may kinetically and thermodynamically affect the phase stability in these materials. To date, this remains a major challenge, both from an experimental and a modelling point of view. Experimentally, it remains very difficult to isolate the crystal size effect from other confounding effects such as defects or partial activation and dedicated synthesis procedures are necessary to obtain crystals in a very narrow size range. From a computational point of view, the challenges are even more formidable, as this requires modelling finite nanocrystals with dimensions comparable to experimental crystal sizes in order to go beyond the artificial cooperativity introduced by periodic boundary conditions (PBCs) and to include possible surface effects, which remains out of reach for the moment (*vide infra*). Recently, Keupp et al. embarked on this journey by investigating how the crystal size of DMOF-1 nanocrystallites, with a critical dimension of up to 13 nm, affects the phase transition in this material (Keupp and Schmid, 2019) (see *Simulation Approaches to Treat the Effect of Crystal Size* for more details). They demonstrated that phase transitions preferentially occur at the crystal surface. For practical applications, it remains a challenge to upscale these observations to a broad range of SPCs and to finite crystallites with critical dimensions of a few hundreds of nanometers and several hundreds of millions of atoms.

## Simulation Approaches to Treat the Effect of Crystal Size

Often the discrepancy between results derived by simulation and experimental observations is bluntly attributed to simulations considering a “perfect” and “infinite” model that can be at odds with materials consisting of discrete crystals with disorder. The extraordinary ability for simulations to capture microscopic differences in materials, such as electronic effects, can render the important macroscopic features of crystal size and morphology out of sight and thus out of scope for many computational studies. Recently, efforts to bridge these disparate length scales have produced ground-breaking simulations and methodology to capture these features and reveal their microscopic influence.

Atomistic simulations can be employed to treat three-dimensional solids, like that of crystalline materials, by using PBCs. In this approach, a three-dimensional boundary is chosen to contain a primitive cell, containing particles that are repeated to construct an infinite solid. The periodic boundary could also be defined in two dimensions for layer systems. This powerful approach permits the efficient computation of the properties of crystalline materials, such as gas adsorption, diffusion and mechanical properties, by considering only repeating units of hundreds of atoms. Thus to consider the discrete nature of crystallites this efficient approach cannot be used and instead the simulation must treat the solid as a large molecule—every atom in the crystallites must be treated. As one can imagine this can become computational unreasonable, for example a single grain of salt has 10^18^ atoms far exceeding the largest systems simulated today, in 2020 a simulation of 137 billion silicon atoms was reported (Hou et al., 2020). Nevertheless, the computation of crystalline materials with the removal of PBCs is necessary to investigate the microscopic origins for crystal size and morphology effects. A range of crystal sizes may be out of reach for current simulation methodologies but a series of nanocrystallites were investigated by the group of Schmid (Keupp and Schmid, 2019). An infinite crystal of DMOF-1 was simulated using PBCs for a unit cell of 216 atoms and compared to nanocrystallites featuring 3 × 3 × 3 to 16 × 16 × 16 unit cells (232,448 atoms). DMOF-1 was investigated as a model system for volumetric phase transformations between an open pore (op) and closed pore (cp) phase. Despite featuring a quarter million atoms, the largest investigated crystallite is still significantly smaller than real systems, for example the 16 × 16 × 16 unit cells system has a diagonal distance of 23.3 nm. However, the important features of the op-to-cp phase transformation can be captured by these simulations and importantly the discrete crystallite systems show surface effects that impede a transition. The value of the free energy difference between the two phases are comparable for an infinite system, using PBCs, and crystallites of 16 × 16 × 16 unit cells. Importantly, only by employing simulations of discrete, aperiodic systems, one can compute meaningful activation barriers and as a result the resulting kinetics of phase transformations. In parallel, thermodynamic insight in the size-dependent switching behaviour of SPCs was obtained by investigating how the crystal size affects the free energy transition barrier (Rogge et al., 2019). While this study was limited by the use of PBCs, investigating mesosized crystals allowed to hypothesize that the transition barrier decreases with increasing crystal size due to the occurrence of phase coexistence, which would explain the experimentally observed rigidity of smaller crystals.

The simulation of large system sizes is crucial for biomolecular systems, such as proteins, which can require the simulation of systems containing millions of atoms. To make these systems computationally feasible, approaches to simplify the computations and approximate atomic systems are used. One of these techniques, used throughout biomolecular and polymer simulations, is coarse-graining where systems are represented not by atoms, but instead using “pseudo-atoms” that approximate groups of atoms, for example a benzene ring. The most common approach to choose pseudo-atoms is the Martini approach, where four non-hydrogen atoms are represented by a single pseudo atom (Marrink et al., 2007). However, the Martini approach has several noted drawbacks (Souza et al., 2020), especially when applied to MOF frameworks that contain highly symmetric or infinitely long chain structures as building units and possible disorder (Cheetham et al., 2016), which prove it difficult to apply directly to framework structures. Nonetheless, Dürholt et al. reported a proof of concept approach of a parameterised coarse grained model for a Cu paddlewheel based MOF, HKUST-1 (Dürholt et al., 2016). A minimal representation was modelled as to reproduce specific low energy deformation modes of the Cu paddlewheel unit and approximate non-bonding interactions. This resulted in the semi-quantitative prediction of key structural and mechanical properties of HKUST-1 using even the maximally coarse grain model. It is noted, however, that this approach is unable to recreate the negative thermal expansion (NTE) present in HKUST-1 (Schneider et al., 2019). The computational savings of this coarse grained approach were leveraged to simulate the local deformation dynamics of nano-indentation using a 5 × 5 × 6 supercell slab model of HKUST-1, which is tens of nanometers in dimension. Recently, Rogge described a micromechanical model to coarse-grain framework structures using nanocells to approximate the particular nodes present in mesoscopic MOF crystals (Rogge, 2021). This approach can drastically reduce the number of degrees of freedom considered, two to three orders of magnitude, and demonstrates how the motion of the unstable and bistable materials can be described. The above coarse-graining methods are important to extend simulations to large unit cells to enable the investigation of disorder like that observed in UiO-66 frameworks (Johnstone et al., 2020).

It is expected that with further increases in computational power and the approaches described above using atomistic simulation methods to investigate the effects of crystal size, and other morphological features, will be possible. In particular, graphical processing units (GPUs) and deep neural network models have the ability to drive molecular dynamics simulations with ab initio accuracy well beyond the performance of conventional simulations (Lu et al., 2021). Only by using these new approaches to simulation methods can the length scale of crystals be efficiently studied.

## The Importance of Crystal Size and Morphology in Drug Delivery

Due to the outstanding properties of MOFs such as high porosity, attractive surface chemistry, and tailorable structure and composition (including utilizing endogenous and/or bioactive motifs to form bioMOFs, Rojas et al., 2017), MOFs have been considered as one of the most promising materials for drug delivery applications (Horcajada et al., 2012; Yang and Yang, 2020; Osterrieth and Fairen-Jimenez, 2021). In drug delivery, the MOF particle size and morphology do not impact only the material physicochemical properties (including the material chemical stability, and drug adsorption and diffusion processes), but also the pharmacokinetics. In nanomedicine, it is widely accepted that the carrier characteristics such as size, morphology and surface properties play a crucial role in the delivery process by influencing the carrier biodistribution, cell internalization as well as its clearance (Mitchell et al., 2021). But what is the ideal carrier size for drug delivery applications? And which particle morphology is the best? There are no simple answers to these questions, because the optimal parameters strongly depend on the particular application and route of administration. However, there is some guidance, which one can follow.

It is largely believed that if drug carriers are distributed intravenously, they should be larger than ca. 10 nm to prevent their fast elimination by renal excretion, and smaller than 200 nm to prevent rapid mononuclear phagocyte recognition (Hoshyar et al., 2016). Additionally, to escape from the circulation through openings (called fenestrations) of the endothelial barrier, nanoparticles should be smaller than 150 nm (Blanco et al., 2015), or in case of tumor targeting, below 200 nm (because around tissues with pathological conditions, discontinuous endothelium with large fenestrations up to 800 nm are often found) (Fang et al., 2011). In transdermal drug delivery, the particle size also matters and influences the depth of the carrier penetration into the skin. For example, it has been reported that particles from around 300 nm to 1.5 µm could pass the stratum corneum via hair follicles, while smaller nanoparticles likely accumulated within the follicle openings (Lademann et al., 2008). Similarly to the particle size, also the particle morphology is important. It has been shown that it can influence the nanocarrier cellular uptake via endocytosis (Rennick et al., 2021). Endocytosis has two stages: adhesion and internalization. The shape of nanoparticles is considered to be important especially for the cell adhesion process. However, when investigating the relationship between the shape of the nanoparticles and cellular uptake, there have been some inconsistent and contrary findings (Wang et al., 2020; Sousa De Almeida et al., 2021). In some studies, spherical nanoparticles were found to be better suited for entering cells than rod-shape nanoparticles, whereas, in other studies, the results were opposite. This indicates that the cellular uptake is a complex process, which is influenced not only by the particle size and morphology, but also by other parameters such as surface chemistry and cell type.

**FIGURE 2:**
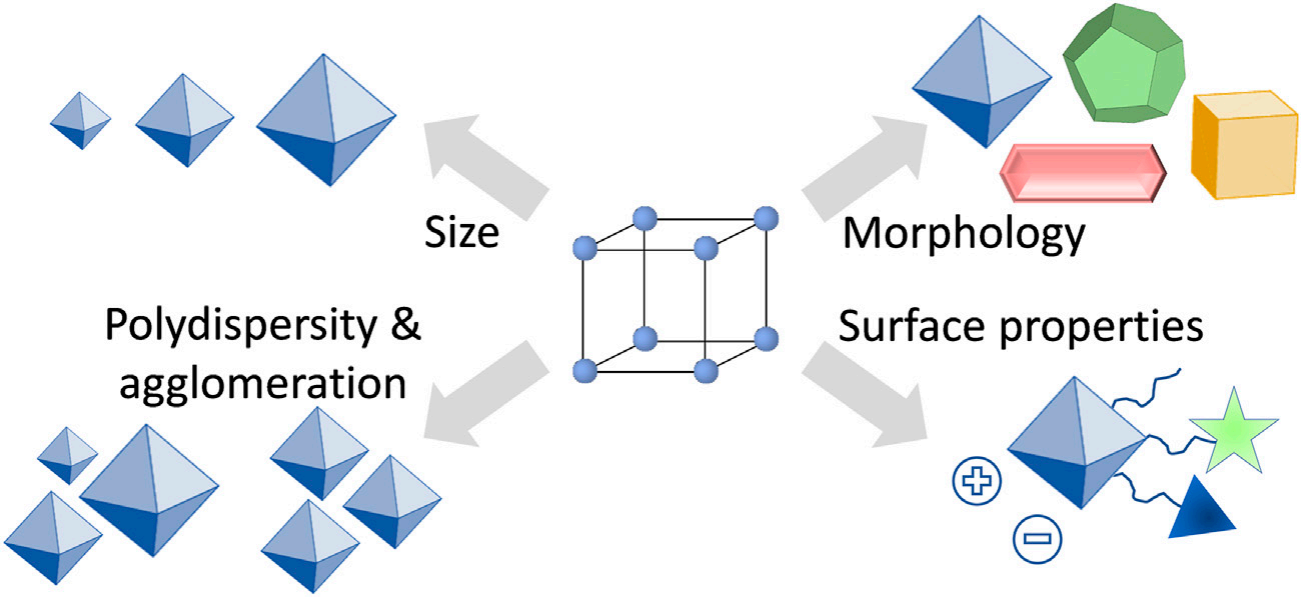
Overview of important characteristics of MOF nanoparticles for applications in drug delivery.

One of the reasons why the understanding of how the size and shape of nanoparticles affect biological systems is far from being complete, is the lack of suitable materials that can be prepared in various, but precisely defined nanoparticle formulations. From this point of view, MOFs seem to be the perfect candidates to fulfil the task, because by altering the synthesis conditions, the characteristics of MOF particles such as size and morphology can be fine-tuned (Stock and Biswas, 2012; Feng et al., 2019; Marshall et al., 2019). However, despite having the possibilities to fine-tune the properties of MOFs (Figure 2), there is so far only very little known about the influence of particle size and morphology on the drug delivery process. In studies using MOFs as nanocarriers, usually spherical particles have been investigated, with some exceptions such as prolonged rod-like crystals of MIL-88A (Horcajada et al., 2010). In most of the studies, only one size of MOF nanocarriers has been examined, usually with an average particle size around or below 200 nm (Horcajada et al., 2012; Yang and Yang, 2020; Osterrieth and Fairen-Jimenez, 2021). There are only very few studies in which different particle sizes of the same material have been tested. For instance, Liu et al. studied on an example of AZIF-8 (amorphous zeolitic imidazolate framework-8) the effects of particle size on the treatment of tumors (Duan et al., 2018). Through a series of *in vitro* and *in vivo* studies, the authors could show that AZIF-8 of 60 nm large nanoparticles had the best curative effects and the highest tumor uptake. In another work, Zhou et al. studied nanoparticles of a MOF PCN-224 for enhanced photodynamic therapy (Park et al., 2016). They reported that PCN-224 with a particle size of 90 nm loaded with a photosensitizer were better performing in photodynamic therapy than PCN-224 with a particle size of 190 nm. Moreover, since MOFs are considered to be intrinsically biodegradable, the particle size influences also the overall carrier stability (Bunzen, 2021). For instance, Falcaro et al. reported on the chemical stability of ZIF-8 with regard to the particle size (Velásquez-Hernández et al., 2019). They showed that smaller crystals (250 nm) decomposed faster in simulated body conditions than larger crystals (2 µm). Last but not least, also surface properties of the nanocarrier are important for the outcome of the drug delivery process (Mitchell et al., 2021; Osterrieth and Fairen-Jimenez, 2021). Therefore, these should be considered and studied in detail together with the particle size and morphology.

There is no doubt that MOFs are highly promising materials for applications in drug delivery. However, despite the fact that there have been already several investigations utilising MOFs as drug nanocarriers carried out *in vivo* (Baati et al., 2013; Simon-Yarza et al., 2016; Sifaoui et al., 2021), systematic studies focused on the influence of the particle size and morphology on the drug delivery process (both *in vitro* and *in vivo*) are still very rare. The lack of such studies is quite surprising considering the unique possibilities of fine-tuning the MOF properties and the accessibility to prepare MOF particles of different sizes and morphologies. Hopefully, this will change in the near future, because the information about the carrier design gained from such studies is essential to boost the field of nanomedicine and to facilitate the transition of nanodrugs from bench to bedside.

## Summary

Clearly, there has been tremendous progress in investigating crystal size and morphology of porous materials and we have outlined here how this can affect mechanical properties, adsorption-induced stress, phase stability and applications in drug delivery, and how computational methods can be employed to provide important insight. These investigations are mostly in their early stages thus clearly illustrating the open challenge in this area and the opportunity for further understanding the effect of crystal size and morphology. Considering these individual fields it becomes obvious that some of them are intrinsically interconnected. In particular adsorption-induced structural transitions in flexible porous materials exhibit features that are covered in chapter 2, 3, and 4 from different point of views. For example, reduction in crystal size is found to reduce adsorption stress, increase mechanical stiffness and enhance the barrier for phase transition. Consequently, individual contributions need to be deciphered impact on all aspects that dictate such responsive behaviour. A more global perspective and intercorrelated analysis of these factors might result in unified principles applicable for other aspects not covered in this work. We believe that these challenges can only be met by a diverse assembly of scientists from unique backgrounds and complementary fields and are excited for the new developments in this area.

## Data Availability

The original contributions presented in the study are included in the article, further inquiries can be directed to the corresponding author.
